# The Low Glutamate Diet Effectively Improves Pain and Other Symptoms of Gulf War Illness

**DOI:** 10.3390/nu12092593

**Published:** 2020-08-26

**Authors:** Kathleen F. Holton, Anna E. Kirkland, Michael Baron, Shalini S. Ramachandra, Mackenzie T. Langan, Elizabeth T. Brandley, James N. Baraniuk

**Affiliations:** 1 Department of Health Studies, American University, Washington, DC 20016, USA; sr6890a@student.american.edu (S.S.R.); brandley@american.edu (E.T.B.); 2 Center for Behavioral Neuroscience, American University, Washington, DC 20016, USA; 3 Program in Behavior, Cognition and Neuroscience, American University, Washington, DC 20016, USA; ak0698a@american.edu; 4 Department of Mathematics & Statistics, American University, Washington, DC 20016, USA; baron@american.edu; 5 Neuroscience Program, Department of Biology, American University, Washington, DC 20016, USA; ml2323a@student.american.edu; 6 Department of Medicine, Georgetown University, Washington, DC 20057, USA; baraniuj@georgetown.edu

**Keywords:** Gulf War Illness, GWI, glutamate, diet, pain, fatigue, symptoms, treatment

## Abstract

Gulf War Illness (GWI) is a multisymptom disorder including widespread chronic pain, fatigue and gastrointestinal problems. The objective of this study was to examine the low glutamate diet as a treatment for GWI. Forty veterans with GWI were recruited from across the US. Outcomes included symptom score, myalgic score, tender point count, dolorimetry and the Chalder Fatigue Scale. Subjects were randomized to the low glutamate diet or a wait-listed control group, with symptom score being compared after one month. Subjects then went onto a double-blind, placebo-controlled crossover challenge with monosodium glutamate (MSG)/placebo to test for return of symptoms. Symptom score was compared between diet intervention and wait-listed controls with an independent t-test and effect size was calculated with Cohen’s *d*. Change scores were analyzed with Wilcoxon Signed Rank tests. Crossover challenge results were analyzed with General Linear Models and cluster analysis. The diet intervention group reported significantly less symptoms (*p* = 0.0009) than wait-listed controls, with a very large effect size, *d* = 1.16. Significant improvements in average dolorimetry (*p* = 0.0006), symptom score, tender point number, myalgic score and the Chalder Fatigue Scale (all *p* < 0.0001) were observed after the 1-month diet. Challenge with MSG/placebo resulted in significant variability in individual response. These results suggest that the low glutamate diet can effectively reduce overall symptoms, pain and fatigue in GWI, but differential results upon challenge suggest that other aspects of the diet, or underlying differences within the population, may be driving these changes. Future research is needed to identify potential nutrient effects, biomarkers, and underlying metabolic differences between responders and non-responders.

## 1. Introduction

Gulf War Illness (GWI) is a multisymptom disorder common to veterans of the first Gulf War (GW). It includes symptoms such as widespread chronic pain, fatigue, cognitive dysfunction, sleep issues and gastrointestinal problems [1,2]. Veterans experiencing GWI have reported an overall lower quality of life as compared to Gulf War veterans who do not have the disorder [3]. Currently, no treatment has been found to result in an improvement in all of the symptoms of GWI [4]. This lack of treatment has resulted in the use of many medications to treat individual symptoms, with little overall improvement for the veterans.

While the etiology of GWI is unknown, it is thought to be connected to central nervous system (CNS) dysfunction that began during the Gulf War due to various neurotoxic exposures such as chemical warfare agents, pyridostigmine bromide (PB) pills, pesticides, burning oil fields and depleted uranium [5,6]. Nerve agents, PB pills and pesticides are all acetylcholinesterase (AChE) inhibitors. When AChE is inhibited, it prevents the breakdown of acetylcholine (ACh) in the synapse which leads to the overactivation of the ACh receptors, and can result in deleterious effects ranging from increased neuronal hyper-excitability to death from seizures or respiratory paralysis [7,8]. However, ACh dysregulation alone does not account for symptoms of GWI [9]. ACh dysregulation has downstream effects on glutamate (Glu), the most ubiquitous excitatory neurotransmitter in the CNS, which results in glutamatergic excitotoxicity, mainly through the overactivation of the N-methyl-D-aspartate (NMDA) ionotropic glutamate receptor [10]. Glutamatergic excitotoxicity triggers neuroinflammation and oxidative stress contributing to increased blood–brain barrier (BBB) permeability and neuronal cell death [8]. The cascade of CNS dysfunction from neurotoxic exposures mediated by glutamatergic excitotoxicity has been proposed as a potential mechanism for GWI and is reviewed in detail elsewhere [11].

One of the most common symptoms of GWI is widespread chronic pain. Glutamate receptors are found all along the pain pathway, including the peripheral nervous system (PNS), the spinal column, and pain processing areas of the brain. Therefore, glutamate plays a critical role in normal pain transmission and abnormal pain sensitization [12]. Central sensitization is the proposed mechanism for widespread chronic pain and is characterized by a neuroplastic shift within the CNS to increase response to both painful and non-painful stimuli. Central sensitization is driven by changes in glutamatergic neurotransmission within the spine (as well as changes in other neuropeptides, such as substance P) which impacts the magnitude and frequency of pain signals sent to the brain [13]. As seen in excitotoxicity, the NMDA receptor is heavily implicated in the initiation and maintenance of central sensitization [14]. Glutamate concentrations have been shown to be increased in pain processing areas of the brain in individuals with fibromyalgia, which has a symptom profile that almost completely overlaps with GWI [15].

Outside of chronic pain, dysregulation of glutamatergic neurotransmission has been implicated as a potential underpinning for many other symptoms of GWI. Glutamate receptors are found throughout the central and peripheral nervous systems, on immune cells, and on many organs (e.g., pancreas, heart, kidney, lungs, skin, gastrointestinal tract) [16]. Therefore, the wide-ranging location of glutamate receptors may contribute to the diverse symptom presentation within GWI. Furthermore, glutamate mediated central sensitization is associated with multisymptom illnesses, which include symptoms such as migraine, cognitive dysfunction, chronic fatigue, and even gastrointestinal issues [17]. Together, it seems as though glutamate dysregulation may be a vital component of GWI.

Glutamate is both an excitatory neurotransmitter and a non-essential dietary amino acid found in both bound forms (e.g., full protein such as meat) and free forms (e.g., food additives including monosodium glutamate (MSG)). In healthy humans, glutamate is actively transported at the BBB and blood-cerebrospinal fluid (CSF) barrier, limiting the amount of dietary glutamate that can enter into the CNS [18]. Permeability of these barriers has been shown to be increased after central sensitization, stress, infection, trauma, and neurotoxic exposures [19,20,21,22]; all events which have been associated with multisymptom disorders, like GWI. This permeability of the BBB and blood–CSF barrier may allow dietary glutamate to enter the CNS in higher amounts.

The low glutamate diet has been proposed as a treatment for multisymptom illness as it eliminates dietary amino acids (e.g., free glutamate and aspartate) that can act on NMDA receptors and contribute to long-term excitotoxicity. Research using the low glutamate diet has shown promising findings both in case studies of fibromyalgia [23] and in a double-blind placebo-controlled crossover challenge study in individuals with fibromyalgia and irritable bowel syndrome [24]. The latter study, completed by our research group, observed >30% remittance of symptoms among 84% of subjects after one month on the low glutamate diet, as well as a significant return of symptoms when challenged with 5 g of MSG over three days relative to placebo. Due to the symptom overlap between fibromyalgia and GWI, the low glutamate diet may be a viable treatment option to help combat many of the symptoms of GWI.

Therefore, the objective of this study was to examine the effectiveness of the low glutamate diet as a treatment for GWI. Specifically, the current study aimed to assess the effects of the low glutamate diet on overall symptom number, pain, and fatigue.

## 2. Materials and Methods

This study was approved by the Institutional Review Boards at American University (IRB# 2017-301) and Georgetown University (IRB# 2017-0811), as well as by the Human Research Protection Office (HRPO) of the US Army Medical Research and Materiel Command (HRPO Log Number A-20203.a). This clinical trial was registered through ClinicalTrials.gov NCT#03342482. All subjects provided written informed consent before participating.

Forty-six veterans of the GW with symptoms meeting both the Kansas criteria [2] and the CDC criteria [1] for GWI were recruited from across the United States. To be eligible, veterans had to fulfill the following inclusion/exclusion criteria as detailed in Table 1.

**Figure 1:**
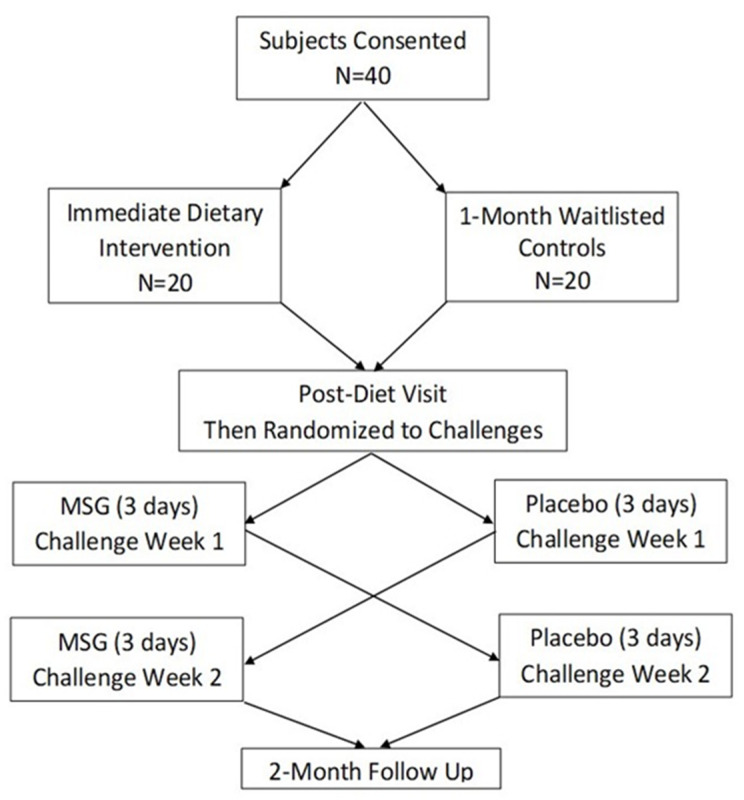
Study design. MSG = monosodium glutamate.

After consent, 6 subjects dropped out, 3 before starting the intervention, and then 3 others during the intervention, (due to: car accident, kidney failure, gastric bypass), as detailed in the Consort Diagram (Appendix A). All subjects traveled to Washington DC for a baseline visit (1 day) and for a post-diet visit plus challenge testing (2 weeks). Figure 1 illustrates the study design.

At the first visit, all baseline measures were assessed, including demographics, anthropometric measurements, heart rate, blood pressure, symptom score, and Chalder Fatigue Scale; and a tender point exam was also completed. The latter resulted in a tender point count, myalgic score and dolorimetry measurements in kilograms over the 18 tender point sites. These dolorimetry measurements were averaged over the 18 sites to give a mean (SD) dolorimetry measurement which could be statistically evaluated. This measure represents the average kilograms of pressure a person can withstand before feeling pain.

After baseline assessment, subjects were randomized to the diet intervention group (where they started the low glutamate diet right away) or to a wait-listed control group (where they started the diet one month later). Total symptom score (as the main outcome) was assessed at the end of this one-month period to assess treatment effects between active intervention and control groups, and to calculate effect size. After completion of the waitlisted control period, these subjects were then put on the low glutamate diet so that everyone had access to the intervention. Once all subjects completed the one-month dietary intervention (described below), pre–post change scores were evaluated to assess the quantification of improvement in all measures.

### 2.1. Low Glutamate Dietary Intervention Training

The low glutamate diet is a healthy, whole food diet that restricts the consumption of free glutamate. As described above, glutamate occurs in the diet in both bound and free forms. Free forms of glutamate can be found as flavor enhancing food additives, as well as in some naturally occurring sources such as soy sauce, aged cheeses, seaweed, mushrooms, and tomato sauce. Free forms of aspartate are also restricted on the diet due to aspartate’s ability to activate the NMDA receptor. Aspartate is found in the artificial sweetener aspartame, hydrolyzed proteins, and gelatin. The diet also optimizes the consumption of specific nutrients known to protect against glutamate excitotoxicity, including vitamin C, magnesium, vitamin D, and omega-3 fatty acids. Participants were also taught how to maximize their consumption of antioxidants to counter the oxidative stress caused by glutamate excitotoxicity. This nutrient optimization was achieved through the consumption of whole food, except for vitamin D and omega-3 fatty acids, which subjects received through the addition of high vitamin D liquid cod liver oil to the diet. Liquid cod liver oil was used to avoid consumption of gelatin capsules.

Participants went through in-depth dietary training with the principal investigator, via Skype. All subjects were provided a binder of materials which included a list of food additives to avoid, foods which commonly contain these additives (including hidden sources), a list of foods highest in each micronutrient, a high antioxidant foods list, a shopping list and sample recipes. Questions which arose about the diet during participation were answered via text. Participants followed the low glutamate dietary intervention from initiation of the diet after baseline assessment until the completion of the last challenge week (~1.5 months total).

### 2.2. Double-Blind Placebo-Controlled Crossover Challenge

After completion of the one-month dietary period, subjects were randomized into the double-blind, placebo-controlled crossover challenge and were instructed to continue following the diet. After an overnight fast, participants went to the lab in the morning, and received pills containing either MSG or placebo, depending on randomization. The challenge pills were identical and were made by an un-blinded research assistant (who was not involved in the collection of any outcome measures) using veggie capsules (to prevent exposure to gelatin). The MSG pills contained 5 g of MSG, whereas the placebo pills contained 5 g of sugar and salt, with the amount of sodium matched to the amount present in MSG. Subjects were monitored for two hours post-consumption for adverse effects. The challenge occurred over three consecutive mornings and outcome measures were re-assessed on the third day. After a one-week wash-out period, subjects returned to the lab to receive the opposite contingency over three consecutive days, again with outcome measures assessed on the third day.

### 2.3. Outcome Measures

The outcome measures used in this study included a total symptom score (the main outcome), as well as secondary more detailed measures of pain using a tender point exam (tender point number, myalgic score, and an average dolorimetry measure taken at the 18 tender point sites) and the Chalder Fatigue Scale as a measure of fatigue severity. The total symptom score was a simple additive measure with a score from 0–33, which captures the total number of symptoms being experienced. It consists of typical GWI symptoms plus “other” as a 33rd response option, where they can write in any additional symptoms that were not included on the questionnaire. Symptom severity was also ranked on a scale of 0–4 for key symptoms to illustrate whether symptom severity improved when a symptom did not remit completely. The Patient Global Impression of Change (PGIC) Scale was used to assess each subject’s perception of improvement on the diet. Dietary compliance was assessed using a glutamate food frequency questionnaire (FFQ) specifically designed for research on the low glutamate diet, and was collected at each assessment period (baseline, post-diet, and the end of each challenge week). A follow-up survey was also sent out to all participants ~3 months after study completion to quantify how many veterans continued to follow the diet post-study completion.

### 2.4. Statistical Methods

All data were double entered into Excel and cross-checked for accuracy. Analyses were conducted using SAS 9.4. The distribution of all continuous variables was assessed using the Shapiro–Wilk test of normality. A comparison of symptom scores between the immediate dietary intervention group and the wait-listed control group was completed using an independent t-test, and treatment effect size was calculated using Cohen’s *d*. Then this measure was compared to the recommendations for a small, medium, large, or very large effect size as described by Sawilowsky [25]. After all participants completed the one-month dietary period, pre–post change scores were assessed for all subjects using the Wilcoxon Signed-Rank test. Subjects were considered to have “improved” on the diet based on two sets of criteria: (1) if they reported to be “much” or “very much” improved on the PGIC, or (2) if they had ≥30% of their symptoms remit after one month on the diet. Bivariate analyses were used to analyze demographic variables according to improvement using Chi-square or Fisher’s Exact tests for categorical variables, and paired t-tests or Wilcoxon Rank Sum tests for continuous variables, depending on normality. Residuals for all dependent variables were normal according to the Shapiro–Wilk test of normality, so general linear modeling (GLM) was used to analyze data from the double-blind, placebo-controlled crossover challenge. Outcomes assessed were total symptom score, number of tender points, total myalgic score, average dolorimetry, and the Chalder Fatigue Scale, each as the dependent variable, and sequence (placebo/MSG, MSG/placebo), period (challenge week 1 v. challenge week 2), challenge material (MSG v. placebo), and subject variability nested within each sequence (SEQ(ID)) as the independent variables. K-means clustering was then used to cluster responses to all 5 outcome measures, after data was normalized, and leave-one-out cross-validation was used to determine the best cluster solution. The Bonferroni correction was used to account for multiple comparisons, with the adjusted significance level of α = 0.01 per each test, guaranteeing the familywise error rate of 0.05.

## 3. Results

Of the forty subjects who completed this study, more than a quarter of the sample was female, the average age was 54 years old, the average BMI was considered obese (i.e., BMI ≥30 kg/m^2^) and 8% were African American. The veterans stemmed from four branches of the military (Army, Airforce, Marines, Navy), the majority were married, and approximately 39% were retired or disabled due to their illness. Table 2 details the demographic statistics.

At baseline, those randomized to the intervention or wait-listed control groups did not significantly differ from one another based on their overall symptom score. After one month, the low glutamate diet intervention group experienced a significant reduction in overall symptoms as compared to the wait-listed control group, with mean (SD) symptom scores of 11.7 (5.3) for the intervention group and 18.1 (5.7) for the wait-listed controls, *p* = 0.0009. This corresponds to an effect size of *d* = 1.16, which is considered a ‘very large’ effect size for the low glutamate diet [25].

A total of 73% of participants improved on the diet based on the PGIC measure, and 65% of participants were considered improved when using the criteria of ≥30% of symptoms remitting after one-month on the diet. Descriptive characteristics for those who improved on the diet are listed in Table 3 in two ways—based on the PGIC measure and based on symptom remission. No significant differences were noted for any of the variables including age, sex, race, education, military rank, or any previous diagnoses when evaluating improvement based on the PGIC; however, those with bronchitis, emphysema or chronic obstructive pulmonary disorder (COPD) were less likely to experience ≥30% symptom remission than those without one of these diagnoses. Those who did and did not improve on the diet reported similar intake of glutamate at baseline, based on the glutamate FFQ, but the post-diet assessment of this compliance measure suggests that those who were not following the diet as well at the post-diet assessment were slightly less likely (with a marginal *p*-value of 0.06) to report remission of ≥30% of symptoms. Similarly, those with chronic fatigue syndrome were marginally less likely to report this level of symptom remission.

Significant reductions were noted in all outcome measures after all subjects completed one month on the diet. These improvements are shown in Table 4. Importantly, zero side effects were reported during the dietary intervention.

**Figure 2:**
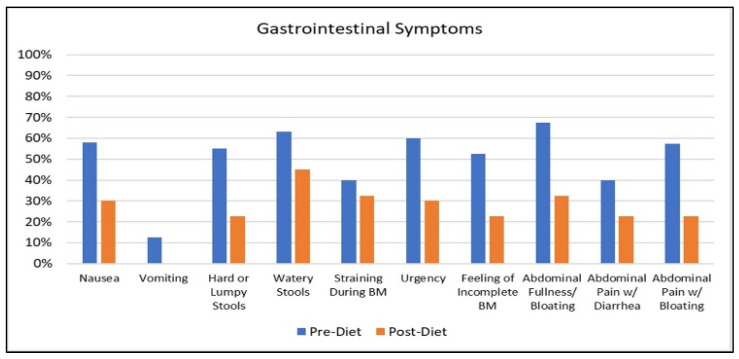
Percentage of veterans reporting gastrointestinal symptoms pre–post diet.

To visualize the frequency of subjects reporting each symptom pre–post diet, graphs were created for the symptoms which composed the symptom score measure. Figure 2 shows the percentage of GW veterans reporting gastrointestinal symptoms at baseline and post-diet. Reductions were seen in all the gastrointestinal symptoms, including reductions in abdominal pain, after the dietary intervention.

**Figure 3:**
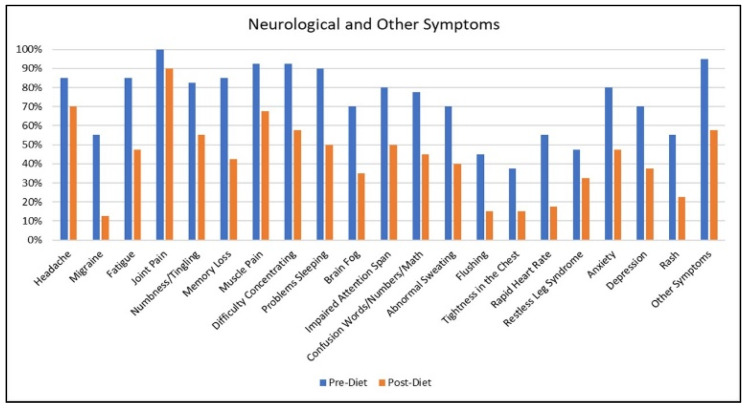
The percentage of veterans reporting neurological and other symptoms pre–post diet.

Figure 3 demonstrates the percentage of veterans reporting neurological and other symptoms, including different types of pain. In comparison to other pain-related symptoms, joint pain showed lesser change. While most veterans reported a reduction in their overall joint pain, those who still reported joint pain post-diet, were more likely to report pain for specific joint(s) that were previously injured. For example, those who were paratroopers commonly reported arthritic pain in their knees and ankles that did not remit on the diet. Conversely, remission of migraine was quite dramatic, with the report of this symptom dropping 42% after one month on the diet. “Other symptoms” in the figure includes report of symptoms such as: PTSD, sleep apnea, tinnitus, muscle twitching, gastroesophageal reflux, temporomandibular joint disorder, shortness of breath and visual disturbances.

**Figure 4:**
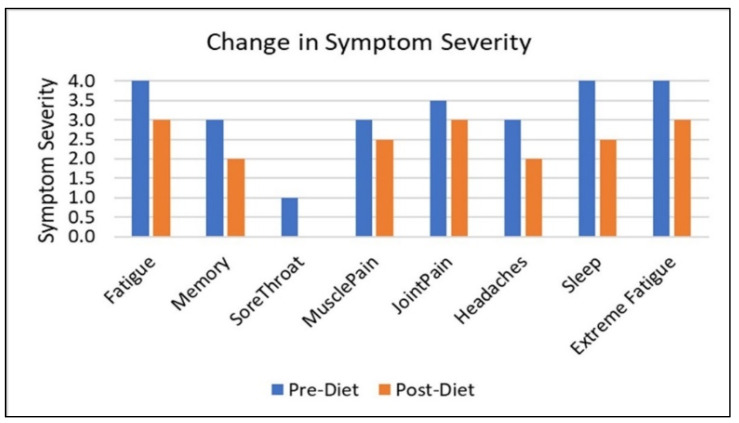
Median symptom severity pre–post diet.

Reductions in the severity of symptoms were also noted after one month on the diet. Subjects did report a reduction in the severity of joint pain even though most did not have this symptom remit completely. Symptom severity changes are illustrated in Figure 4. Since these are not normally distributed, median scores are presented at baseline and post-diet.

The primary outcome, total symptom score, as well as the secondary pain measures and Chalder Fatigue Scale, were assessed for a return of symptoms upon challenge with MSG relative to placebo, using the double-blind, placebo-controlled crossover challenge data. As illustrated below in Table 5, the three models with the pain outcomes were significant; however, these results were driven by a sequence effect and an inter-individual variation within sequence, as opposed to a treatment effect. Comparing the change in scores between the two parts of the crossover study, we concluded that the effect of sequence was due to a significant placebo effect, rather than a carryover effect due to an inadequate washout period (which we effectively ruled out). To better understand this variation of subject response within sequence, cluster analysis was used on the change scores (MSG-placebo) for all five outcome measures. This resulted in a best-fit for a 3-cluster solution, determined by the K-means clustering method and verified by leave-one-out cross-validation, with cluster 1 showing a worsening across all 5 measures upon challenge with MSG relative to placebo, cluster 2 showing an improvement in all 5 measures, and cluster 3 demonstrating very little difference between MSG and placebo challenges. We analyzed these clusters for differences and the only variable which was significantly different across clusters was pre–post change in the glutamate FFQ (as a measure of change in the consumption of glutamate); mean (SD) change in FFQ was −74 (18) for cluster 1, −58 (12) for cluster 2, and −56 (18) for cluster 3, *p* = 0.01.

The follow-up survey was collected from 34 of the 40 study subjects ~3 months after subjects completed their study participation. The surveys demonstrated that 30/34 (88%) of subjects were still following the diet at this time point.

## 4. Discussion

This study assessed the effectiveness of the low glutamate diet as a treatment option for GWI and demonstrated a very large treatment effect, with symptoms significantly remitting after one month on the diet. Using a very conservative definition of improvement (≥30% of symptoms remitting), 65% of subjects were considered improved after one month on the diet; whereas when using the PGIC criteria of “much” or “very much” improved was used, 73% of subjects were considered improved. Pre–post diet changes demonstrated significant improvements in symptom severity, pain measures, and a measure of fatigue. To our knowledge, this is the first study to result in widespread symptom improvement in Gulf War Illness and no study has come close to the very large effect size observed herein. Furthermore, there were zero side effects to this intervention, making it a safer alternative to the many drug options currently being used. The variability in the response to the challenges with MSG vs. placebo could suggest that these sub-sets of veterans may differ in their intake of protective dietary factors, or that they potentially have different metabolic profiles, possibly mediated by energy metabolism. Potential mechanisms for future study will be discussed below.

As we have discussed previously, exposures to certain chemicals in the GW caused indirect release of glutamate mediated by effects on ACh [11]. Some of these exposures have also been shown to increase the likelihood of the BBB becoming permeable [26,27], giving dietary glutamate more access to the brain. High levels of glutamate can lead to excitotoxicity, which is known to cause both oxidative stress and neuroinflammation, leading to cell death [28]. The goal of the low glutamate diet is to reduce dietary exposure to free glutamate, while also increasing intake of nutrients known to be protective against excitotoxicity and oxidative stress. The combination of this approach may modulate the direct effect of dietary glutamate perpetuating excitotoxicity within the CNS via the NMDA receptor, and/or help counter the downstream effects of oxidative stress with high antioxidant intake. While this study demonstrated drastic improvements in health from the low glutamate diet, the variability of reaction to MSG relative to placebo in the challenge period suggests that removal of direct exposure to dietary glutamate is not the sole mechanism for these dietary effects when examining symptoms of GWI. These results differ from our previous work in fibromyalgia, where subjects not only improved on the diet, but also had an overall significant return of symptoms upon challenge with MSG relative to placebo [24]. One difference between this study and our previous work in fibromyalgia is the inclusion of nutritional training on how to optimize intake of nutrients which are protective against excitotoxicity and oxidative stress. Thus, the variability of response to challenge with MSG in the current study may be due to protective nutrients helping to optimize the handling of glutamate in the nervous system.

It is also possible that the diet is improving neuronal cellular energy metabolism, which not only helps explain the improvements in fatigue (by improving mitochondrial function and ATP production) [29], but also by potentially improving the handling of glutamate throughout the process of neurotransmission [28]. Glutamate must be tightly regulated once released from the pre-synaptic neuron, and it is well known that astrocytic glutamate transporters must be functioning optimally to remove excess glutamate from the synaptic cleft in order to prevent excitotoxicity [30]. This is an energy intensive process in the astrocyte, and improved cellular energy metabolism, through the increased availability of nutrient cofactors, can help optimize this process (or maintain it longer during times of high levels of glutamate) resulting in reduced excitotoxicity [31]. Improved handling of glutamate between the synaptic cleft and astrocyte during neurotransmission would not only reduce excitotoxicity but would also lessen oxidative stress inside the CNS. This result, combined with higher intake of antioxidants from the diet, may be effectively reducing oxidative stress and possibly also reducing neuroinflammation [32]. Thus, these are important future lines of inquiry.

It is important to note that the high rate of veterans continuing to follow the diet after study completion suggests that the diet is being perceived as worthwhile and feasible. This is a very important aspect of any dietary intervention, and if these findings are replicated in a larger clinical trial, this suggests potential wide-spread utility for the diet as a treatment for veterans with GWI.

A strength of this research was the inclusion of subjects from across the US with adequate representation of female veterans (who are not always included in research studies in adequate numbers). However, since the diet was only tested in 40 subjects, it is very important that a phase 3 clinical trial with a larger sample size be completed to confirm these findings in a larger population of veterans.

Further analyses will be needed for this GWI research to analyze both dietary intake and blood measures of nutrients to better understand which aspects of the diet may be driving these results apart from the removal of free glutamate. Future research should also include mechanistic analyses to better understand how the diet is affecting brain function and blood measures, as well as potential metabolic differences which may be driving differential response to challenge with MSG/placebo.

## 5. Conclusions

The low glutamate diet was shown to effectively treat the broad range of symptoms associated with Gulf War Illness with a very large effect size, indicating a strong clinically relevant outcome. After one month on the diet, an average of nine symptoms remitted, and highly significant improvements were also noted in pain and fatigue measures. Challenge with MSG relative to placebo resulted in variable response between individuals which may indicate metabolic differences among sub-groups of veterans. Thus, further research is needed to understand the mechanism of this effect and to confirm these results in a larger group of veterans.

## Figures and Tables

**Table 1:** Inclusion and exclusion criteria for clinical trial.

Inclusion Criteria	Exclusion Criteria
<75 years of age	Active military duty
Stable medication dose/frequency > 3 mos	Substance use disorder (past year)
Active deployment during GW	Current nicotine user
Willing to change diet	Past or current seizure disorder
Willing to discontinue alcohol & marijuana	Severe asthma requiring hospitalization
	Medication affecting Glu neurotransmission

GW = Gulf War.

**Table 2:** Descriptive characteristics of study population at baseline.

Demographics (*n* = 40)	Mean (SD)
Age (yrs)	54.35 (6.02)
BMI (kg/m^2^)	32.10 (5.34)
	***n* (%)**
Sex	
Female	11 (27.5%)
Male	29 (72.5%)
Race	
Caucasian	37 (92.5%)
African American	3 (7.5%)
Military Branch	
Army	20 (51.3%)
Air Force	6 (15.4%)
Navy	5 (12.8%)
Marine Corps	8 (20.5%)
Highest Education	
High School Graduate	3 (7.7%)
Some college	15 (38.5%)
Graduate school	11 (28.2%)
Postgraduate school	10 (25.6%)
Marital Status	
Single (never married)	1 (2.6%)
Married	28 (71.8%)
Divorced/Separated	7 (17.9%)
Widowed	3 (7.7%)
Employment Status	
Currently working	17 (43.6%)
Homemaker	1 (2.6%)
Retired (not due to illness)	4 (10.3%)
Disabled/Retired due to illness	15 (38.5%)
Unemployed	2 (5.1%)
Smoking Status	
Never smoked	26 (65.0%)
Quit for >6 months	13 (32.5%)
Quit for <6 months	1 (2.5%)

**Table 3:** Characteristics of those who improved on the diet based on a report of “much” or “very much” improved on the patient global impression of change scale and based on remission of ≥30% of their symptoms.

*n* = 40	Improvement: Based on PGIC		*p*-Value *	Improvement: ≥30% Symptoms Remitted		*p*-Value *
	No *n* = 11 (27.5%)	Yes *n* = 29 (72.5%)		No *n* = 14 (35%)	Yes *n* = 26 (65%)	
	Number (%)	Number (%)		Number (%)	Number (%)	
Sex						
Female	2 (18%)	9 (82%)	0.69	3 (27%)	8 (73%)	0.72
Male	9 (31%)	20 (69%)		11 (38%)	18 (62%)	
Race						
Caucasian	11 (31%)	25 (69%)	N/A	14 (39%)	22 (61%)	N/A
African American	0 (0%)	3 (100%)		0 (0%)	3 (100%)	
Education						
Some College or Less	5 (28%)	13 (72%)	0.96 **	6 (33%)	12 (67%)	0.76 **
Graduated College or more	6 (29%)	15 (71%)		8 (38%)	13 (62%)	
Highest Military Rank						
Enlisted	9 (30%)	21 (70%)	0.99	11 (37%)	19 (63%)	0.99
Officer or Warrant Officer	2 (22%)	7 (78%)		3 (33%)	6 (67%)	
** Previous Diagnoses**						
Fibromyalgia						
No	1 (8%)	11 (92%)	0.12	2 (17%)	10 (83%)	0.15
Yes	10 (37%)	17 (63%)		12 (44%)	15 (56%)	
Chronic Fatigue Syndrome						
No	1 (9%)	10 (91%)	0.13	1 (9%)	10 (91%)	0.06
Yes	10 (36%)	18 (64%)		13 (46%)	15 (54%)	
Allergic Rhinitis						
No	6 (24%)	19 (76%)	0.44 **	8 (32%)	17 (68%)	0.50**
Yes	5 (36%)	9 (64%)		6 (43%)	8 (57%)	
Sinusitis						
No	6 (27%)	16 (73%)	0.88 **	6 (27%)	16 (73%)	0.20**
Yes	5 (29%)	12 (71%)		8 (47%)	9 (53%)	
Nasal Polyps						
No	10 (28%)	26 (72%)	0.99	13 (36%)	23 (64%)	0.99
Yes	1 (33%)	2 (66%)		1 (33%)	2 (67%)	
Asthma						
No	9 (29%)	22 (71%)	0.99	11 (35%)	20 (65%)	0.99
Yes	2 (25%)	6 (75%)		3 (37%)	5 (63%)	
Depression						
No	4 (40%)	6 (60%)	0.42	4 (40%)	6 (60%)	0.99
Yes	7 (24%)	22 (76%)		10 (34%)	19 (66%)	
Diabetes						
No	11 (34%)	21 (66%)	N/A	13 (41%)	19 (59%)	0.39
Yes	0 (0%)	7 (100%)		1 (14%)	6 (86%)	
Thyroid Disease						
No	9 (24%)	28 (76%)	N/A	12 (32%)	25 (68%)	N/A
Yes	2 (100%)	0 (0%)		2 (100%)	0 (0%)	
Bronchitis/Emphysema/COPD						
No	8 (24%)	26 (76%)	0.13	10 (29%)	24 (71%)	0.05
Yes	3 (60%)	2 (40%)		4 (80%)	1 (20%)	
Heart Disease						
No	10 (27%)	27 (73%)	0.49	13 (35%)	24 (65%)	0.99
Yes	1 (50%)	1 (50%)		1 (50%)	1 (50%)	
High Blood Pressure						
No	8 (38%)	13 (62%)	0.17	10 (48%)	11 (52%)	0.18
Yes	3 (17%)	15 (83%)		4 (22%)	14 (78%)	
Stroke						
No	10 (26%)	28 (74%)	N/A	13 (34%)	25 (66%)	N/A
Yes	1 (100%)	0 (0%)		1 (100%)	0 (0%)	
Acid Reflux, Ulcers, or Stomach/Intestinal Problems						
No	2 (20%)	8 (80%)	0.70	3 (30%)	7 (70%)	0.72
Yes	9 (31%)	20 (69%)		11 (38%)	18 (62%)	
Liver Disease						
No	10 (27%)	27 (73%)	0.49	13 (35%)	24 (65%)	0.99
Yes	1 (50%)	1 (50%)		1 (50%)	1 (50%)	
Kidney Disease						
No	10 (26%)	28 (74%)	N/A	14 (37%)	24 (63%)	N/A
Yes	1 (100%)	0 (0%)		0 (0%)	1 (100%)	
Back Pain: Neck						
No	1 (33%)	2 (67%)	0.99	1 (33%)	2 (67%)	0.99
Yes	10 (28%)	26 (72%)		13 (36%)	23 (64%)	
Back Pain: Middle Back						
No	2 (13%)	14 (87%)	0.09	5 (31%)	11 (69%)	0.61 **
Yes	9 (39%)	14 (61%)		9 (39%)	14 (61%)	
Back Pain: Low						
No	2 (50%)	2 (50%)	0.56	3 (75%)	1 (25%)	0.12
Yes	9 (26%)	26 (74%)		11 (31%)	24 (69%)	
Osteoarthritis, Degenerative Arthritis						
No	6 (30%)	14 (70%)	0.80 **	10 (50%)	10 (50%)	0.10
Yes	5 (26%)	14 (74%)		4 (21%)	15 (79%)	
Rheumatoid Arthritis/ Autoimmune Disorder						
No	8 (28%)	21 (72%)	0.99	11 (38%)	18 (62%)	0.72
Yes	3 (30%)	7 (70%)		3 (30%)	7 (70%)	
Anemia or Blood Diseases						
No	7 (22%)	25 (78%)	0.08	10 (31%)	22 (69%)	0.23
Yes	4 (57%)	3 (43%)		4 (57%)	3 (43%)	
Cancer						
No	10 (29%)	25 (71%)	0.99	12 (34%)	23 (66%)	0.61
Yes	1 (25%)	3 (75%)		2 (50%)	2 (50%)	
	**Median (IQR)**	**Median (IQR)**	*p*-value ***	**Median (IQR)**	**Median (IQR)**	*p*-value ***
Age	53.00 (9)	54.00 (7)	0.34	53.00 (7)	54.00 (8)	0.23
Years in Military	12.00 (16.5)	9.75 (15.3)	0.51	10.00 (14)	10.00 (15)	0.84
Baseline Glutamate FFQ	71.00 (34)	70.00 (16)	0.60	72.00 (32)	71.00 (23)	0.66
Post-diet Glutamate FFQ	7.00 (10)	4.00 (8)	0.36	8.50 (11)	4.00 (7)	0.06
	**Mean (SD)**	**Mean (SD)**	***p*-value †**	**Mean (SD)**	**Mean (SD)**	***p*-value †**
Baseline BMI	32.78 (6)	32.01 (5)	0.68	31.29 (6)	32.54 (5)	0.49
Post-diet BMI	32.36 (5)	31.66 (5)	0.69	30.76 (6)	32.04 (5)	0.46

* Fisher’s Exact except where ** Chi-square; *** Wilcoxon Rank Sum; † Independent t-test; one person was missing data regarding previous diagnoses, race, education, years in military and highest military rank. PGIC = Patient Global Impression of Change; COPD = chronic obstructive pulmonary disorder; IQR = interquartile range; FFQ = food frequency questionnaire

**Table 4:** Change in outcome measures pre–post diet for all subjects.

Outcome Measure	Pre-Diet	Post-Diet	
	**Median (IQR)**	**Median (IQR)**	*p*-value *
Dolorimetry (kgs) * Higher is better	15 (11)	18 (10)	0.0006
	**Mean (SD)**	**Mean (SD)**	*p*-value **
Symptom Score	21 (5)	12 (5)	<0.0001
Tender Point Number	12 (5)	10 (5)	<0.0001
Myalgic Score	22 (12)	14 (9)	<0.0001
Chalder Fatigue Scale	29 (8)	16 (9)	<0.0001

* Wilcoxon Signed Rank or ** paired t-test. All tests were significant at the Bonferroni adjusted significance level of α = 0.01.

**Table 5:** Results of the crossover challenge with MSG relative to placebo.

Measure	Placebo Mean (SD)	MSG Mean (SD)	F-Statistic	Model *p*-Value *
Total Symptom Score	12.00 (6.65)	11.33 (5.88)	1.21	0.28
Number Tender Points	9.62 (5.49)	9.48 (5.49)	5.10	<0.0001
Total Myalgic Score	16.13 (11.78)	15.36 (11.34)	5.08	<0.0001
Average Dolorimetry * higher is better	17.35 (6.16)	11.27 (5.85)	10.31	<0.0001
Chalder Fatigue Scale	16.28 (9.44)	16.55 (8.57)	0.79	0.77

* General Linear Model accounting for sequence, period, challenge material and subject nested within sequence. Bonferroni corrected *p*-value of *p* = 0.01.

**Figure A1:**
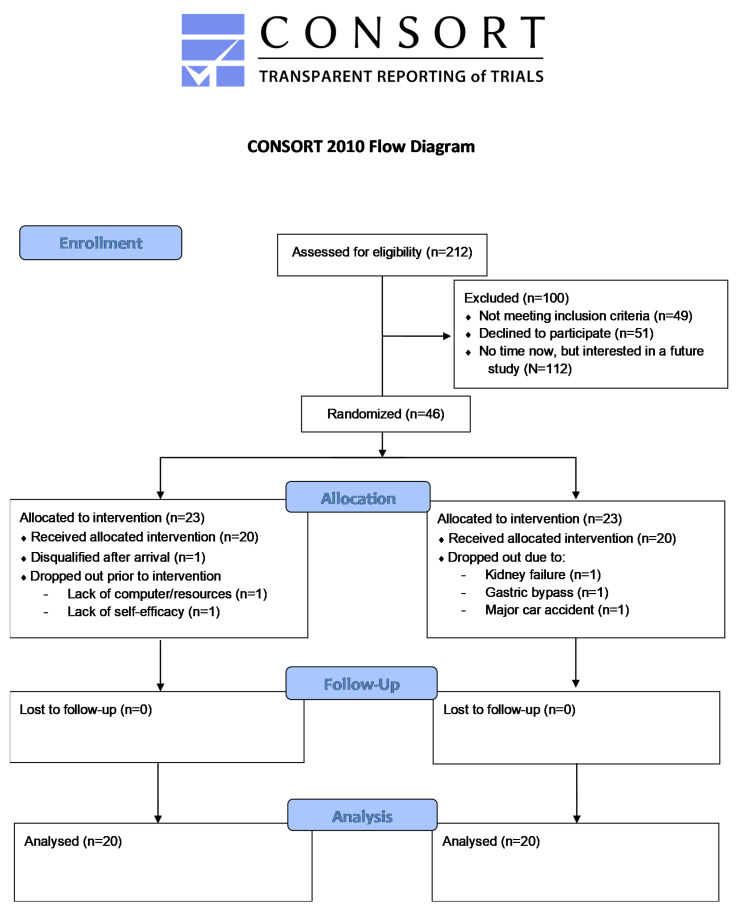
CONSORT diagram reporting the flow of recruitment, allocation, follow-up, and analysis for this clinical trial.
